# Supplementary material for: Resolving the full spectrum of human genome variation using Linked-Reads
Source: Genome Res. 2019 Apr;29(4):635–45. doi: 10.1101/gr.234443.118 (PMC6442396; doi:10.1101/gr.234443.118)
Supplement: Supplemental Material [file supp_gr.234443.118_Supplemental_longranger-2.2.zip › longranger-2.2.tar.bz2!/lr2.2-public/lib/stan/cmdstan/stan/doc/api/html/structstan_1_1gm_1_1generate__local__var__init__nan__visgen.html]

Stan: stan::gm::generate\_local\_var\_init\_nan\_visgen Struct Reference


|  |  |
| --- | --- |
|  | Stan  2.5.0  probability, sampling & optimization |


- Main Page
- Related Pages
- Namespaces
- Classes
- Files

- Class List
- Class Index
- Class Hierarchy
- Class Members

All Classes Namespaces Files Functions Variables Typedefs Enumerations Enumerator Friends Macros Pages

- stan
- gm
- generate\_local\_var\_init\_nan\_visgen

Public Member Functions |
Public Attributes |
List of all members

stan::gm::generate\_local\_var\_init\_nan\_visgen Struct Reference

`#include <generator.hpp>`

Inheritance diagram for stan::gm::generate\_local\_var\_init\_nan\_visgen:

|  |  |
| --- | --- |
| Public Member Functions | |
|  | generate\_local\_var\_init\_nan\_visgen (bool declare\_vars, bool is\_var, bool is\_fun\_return, int indent, std::ostream &o) |
|  | |
| void | operator() (const nil &) const |
|  | |
| void | operator() (const int\_var\_decl &x) const |
|  | |
| void | operator() (const double\_var\_decl &x) const |
|  | |
| void | operator() (const vector\_var\_decl &x) const |
|  | |
| void | operator() (const row\_vector\_var\_decl &x) const |
|  | |
| void | operator() (const matrix\_var\_decl &x) const |
|  | |
| void | operator() (const unit\_vector\_var\_decl &x) const |
|  | |
| void | operator() (const simplex\_var\_decl &x) const |
|  | |
| void | operator() (const ordered\_var\_decl &x) const |
|  | |
| void | operator() (const positive\_ordered\_var\_decl &x) const |
|  | |
| void | operator() (const cholesky\_factor\_var\_decl &x) const |
|  | |
| void | operator() (const cholesky\_corr\_var\_decl &x) const |
|  | |
| void | operator() (const cov\_matrix\_var\_decl &x) const |
|  | |
| void | operator() (const corr\_matrix\_var\_decl &x) const |
|  | |
| template<typename T > | |
| void | generate\_init (const T &x) const |
|  | |
| Public Member Functions inherited from stan::gm::visgen | |
|  | visgen (std::ostream &o) |
|  | |

|  |  |
| --- | --- |
| Public Attributes | |
| const bool | declare\_vars\_ |
|  | |
| const bool | is\_var\_ |
|  | |
| const bool | is\_fun\_return\_ |
|  | |
| const int | indent\_ |
|  | |
| Public Attributes inherited from stan::gm::visgen | |
| std::ostream & | o\_ |
|  | |

|  |  |
| --- | --- |
| Additional Inherited Members | |
| Public Types inherited from stan::gm::visgen | |
| typedef void | result\_type |
|  | |

## Detailed Description

Definition at line 1227 of file generator.hpp.

## Constructor & Destructor Documentation

|  |  |  |  |
| --- | --- | --- | --- |
| stan::gm::generate\_local\_var\_init\_nan\_visgen::generate\_local\_var\_init\_nan\_visgen | ( | bool | *declare\_vars*, |
|  |  | bool | *is\_var*, |
|  |  | bool | *is\_fun\_return*, |
|  |  | int | *indent*, |
|  |  | std::ostream & | *o* |
|  | ) |  |  |

inline

Definition at line 1232 of file generator.hpp.

## Member Function Documentation

template<typename T >

|  |  |  |  |  |  |
| --- | --- | --- | --- | --- | --- |
| void stan::gm::generate\_local\_var\_init\_nan\_visgen::generate\_init | ( | const T & | *x* | ) | const |

inline

Definition at line 1286 of file generator.hpp.

|  |  |  |  |  |  |
| --- | --- | --- | --- | --- | --- |
| void stan::gm::generate\_local\_var\_init\_nan\_visgen::operator() | ( | const nil & |  | ) | const |

inline

Definition at line 1243 of file generator.hpp.

|  |  |  |  |  |  |
| --- | --- | --- | --- | --- | --- |
| void stan::gm::generate\_local\_var\_init\_nan\_visgen::operator() | ( | const int\_var\_decl & | *x* | ) | const |

inline

Definition at line 1246 of file generator.hpp.

|  |  |  |  |  |  |
| --- | --- | --- | --- | --- | --- |
| void stan::gm::generate\_local\_var\_init\_nan\_visgen::operator() | ( | const double\_var\_decl & | *x* | ) | const |

inline

Definition at line 1249 of file generator.hpp.

|  |  |  |  |  |  |
| --- | --- | --- | --- | --- | --- |
| void stan::gm::generate\_local\_var\_init\_nan\_visgen::operator() | ( | const vector\_var\_decl & | *x* | ) | const |

inline

Definition at line 1252 of file generator.hpp.

|  |  |  |  |  |  |
| --- | --- | --- | --- | --- | --- |
| void stan::gm::generate\_local\_var\_init\_nan\_visgen::operator() | ( | const row\_vector\_var\_decl & | *x* | ) | const |

inline

Definition at line 1255 of file generator.hpp.

|  |  |  |  |  |  |
| --- | --- | --- | --- | --- | --- |
| void stan::gm::generate\_local\_var\_init\_nan\_visgen::operator() | ( | const matrix\_var\_decl & | *x* | ) | const |

inline

Definition at line 1258 of file generator.hpp.

|  |  |  |  |  |  |
| --- | --- | --- | --- | --- | --- |
| void stan::gm::generate\_local\_var\_init\_nan\_visgen::operator() | ( | const unit\_vector\_var\_decl & | *x* | ) | const |

inline

Definition at line 1261 of file generator.hpp.

|  |  |  |  |  |  |
| --- | --- | --- | --- | --- | --- |
| void stan::gm::generate\_local\_var\_init\_nan\_visgen::operator() | ( | const simplex\_var\_decl & | *x* | ) | const |

inline

Definition at line 1264 of file generator.hpp.

|  |  |  |  |  |  |
| --- | --- | --- | --- | --- | --- |
| void stan::gm::generate\_local\_var\_init\_nan\_visgen::operator() | ( | const ordered\_var\_decl & | *x* | ) | const |

inline

Definition at line 1267 of file generator.hpp.

|  |  |  |  |  |  |
| --- | --- | --- | --- | --- | --- |
| void stan::gm::generate\_local\_var\_init\_nan\_visgen::operator() | ( | const positive\_ordered\_var\_decl & | *x* | ) | const |

inline

Definition at line 1270 of file generator.hpp.

|  |  |  |  |  |  |
| --- | --- | --- | --- | --- | --- |
| void stan::gm::generate\_local\_var\_init\_nan\_visgen::operator() | ( | const cholesky\_factor\_var\_decl & | *x* | ) | const |

inline

Definition at line 1273 of file generator.hpp.

|  |  |  |  |  |  |
| --- | --- | --- | --- | --- | --- |
| void stan::gm::generate\_local\_var\_init\_nan\_visgen::operator() | ( | const cholesky\_corr\_var\_decl & | *x* | ) | const |

inline

Definition at line 1276 of file generator.hpp.

|  |  |  |  |  |  |
| --- | --- | --- | --- | --- | --- |
| void stan::gm::generate\_local\_var\_init\_nan\_visgen::operator() | ( | const cov\_matrix\_var\_decl & | *x* | ) | const |

inline

Definition at line 1279 of file generator.hpp.

|  |  |  |  |  |  |
| --- | --- | --- | --- | --- | --- |
| void stan::gm::generate\_local\_var\_init\_nan\_visgen::operator() | ( | const corr\_matrix\_var\_decl & | *x* | ) | const |

inline

Definition at line 1282 of file generator.hpp.

## Member Data Documentation

const bool stan::gm::generate\_local\_var\_init\_nan\_visgen::declare\_vars\_

Definition at line 1228 of file generator.hpp.

const int stan::gm::generate\_local\_var\_init\_nan\_visgen::indent\_

Definition at line 1231 of file generator.hpp.

const bool stan::gm::generate\_local\_var\_init\_nan\_visgen::is\_fun\_return\_

Definition at line 1230 of file generator.hpp.

const bool stan::gm::generate\_local\_var\_init\_nan\_visgen::is\_var\_

Definition at line 1229 of file generator.hpp.

---

The documentation for this struct was generated from the following file:

- src/stan/gm/generator.hpp


---

[ Stan Home Page ]

*© 2011–2014,
Stan Development Team.*
